# Prevalence of work-related burnout and associated factors among police officers in the Silesian Voivodeship - the region with the highest urbanization rate and population density in Poland

**DOI:** 10.3389/fpubh.2026.1917062

**Published:** 2026-09-10

**Authors:** Jolanta Malinowska-Borowska, Michał Meiser, Michał Górski

**Affiliations:** 1 Department of Occupational Medicine and Hygiene, Chronic Diseases and Civilization Threats, Faculty of Public Health in Bytom, Medical University of Silesia in Katowice, Katowice, Poland; 2 Student Scientific Circle at the Department of Occupational Medicine and Hygiene, Chronic Diseases and Civilization Threats, Faculty of Public Health in Bytom, Medical University of Silesia in Katowice, Katowice, Poland

**Keywords:** burnout, Link Burnout Questionnaire (LBQ), Poland, police, stress

## Abstract

**Introduction:**

Occupational burnout is one of the most frequently analyzed psychosocial phenomena in the contemporary professional environment, posing a significant challenge for both individuals and entire organizations. The aim of this study was to examine the relationships between the level of occupational burnout and selected psychosocial factors, sociodemographic characteristics, and working conditions among police officers in the Silesian Voivodeship.

**Methods:**

The study was conducted in 2025 on a sample of 363 officers from 24 organizational units. An original survey questionnaire and the standardized Link Burnout Questionnaire (LBQ) were utilized. Data analysis included non-parametric tests, Welch's *t*-test, and a multiple linear regression model.

**Results:**

High levels of psychophysical exhaustion were found in 39.9% of the participants, and disillusionment with work in 49.6%. Pleasure derived from work emerged as the strongest protective predictor against exhaustion (*B* = −4.88; *p* < 0.001). Significant risk factors included poor workplace relationships (*B* = 2.97; *p* < 0.001) and experiences of workplace mobbing (*B* = 1.34; *p* = 0.028). Lack of team support proved to be a powerful stressor (*d* = 0.71). It was also demonstrated that a recovery deficit (only 1 day off per week) strongly increases exhaustion levels (*p* < 0.001).

**Conclusions:**

Occupational burnout among Silesian police officers is strongly determined by relational and organizational factors. Systemic prevention should focus on improving organizational culture, eliminating mobbing, and ensuring adequate time for psychophysical regeneration.

## 1 Introduction

Occupational burnout is one of the most frequently analyzed psychosocial phenomena in the contemporary professional environment, posing a significant challenge for both individuals and entire organizations. This concept was first introduced by Freudenberger (1), who described a state of chronic emotional exhaustion and loss of engagement in work. Further it was developed by Christina Maslach, who defined burnout as a syndrome comprising three co-occurring components: emotional exhaustion, depersonalization, and a reduced sense of personal accomplishment (2). According to the World Health Organization, occupational burnout is a consequence of long-term workplace stress that has not been successfully managed (3). It is not treated as a disease but as a syndrome that affects the professional, emotional, and social functioning of the individual. Notably, occupational burnout often occurs in professional groups requiring constant contact with people, high responsibility, and continuous decision-making. Police officers represent a primary example of such a professional group.

Police officers constitute a professional group of a unique nature. Their service involves performing tasks on the border of law, public safety, and intervention in dangerous situations, which makes this work specifically stressful (4). Officers operate under conditions of high emotional tension, often in the face of aggression, violence, suffer, or death (5). In Poland, police officers are also distinguished by a specific legal status. A distinct list of occupational diseases has been established for them and they are subject to a separate pension system, which includes the possibility of early retirement (6). These regulations reflect the recognition of the arduousness and increased occupational risk resulting from the performance of specific tasks within this professional group. However, in practice, even these solutions do not protect officers from the negative consequences of long-term mental and emotional strain (7).

Work in the Police is not only demanding but also multidimensionally burdensome. Officers perform their service in a shift system, often during night hours, on weekends, and holidays, under conditions of great uncertainty and time pressure. They must react to unpredictable situations, make quick decisions in a dynamically changing environment, and demonstrate mental and emotional resilience when in contact with violence, human tragedies, or life-threatening situations. Research indicates that chronic overload with duties, an excessive pace of work, and constant stress lead to emotional exhaustion - the main component of occupational burnout (8–10). Furthermore, the lack of adequate support from supervisors and colleagues can intensify the sense of isolation, while a shortage of organizational resources (such as equipment, procedures, or training) further exacerbates the psychological burden (10).

High professional demands combined with a lack of adequate benefits - such as additional remuneration, professional promotion, or other forms of recognition - are, in alignment with Siegrist's model (11), among the most important stressors. For police officers, who often undertake risky activities, exceed working time standards, and are constantly assessed by society, such an imbalance between effort and reward fosters the development of cynicism, a sense of lack of meaning in work, and distancing oneself from duties (12). An additional burdensome factor includes unclear organizational structures, conflicting expectations from supervisors, and jurisdictional conflicts between units, which increase emotional tension and frustration (13, 14).

The above-mentioned factors are of particular importance in the context of the Silesian Voivodeship - the region with the highest urbanization rate and population density in Poland. Silesia, as an area of strong industrialization and high socio-economic dynamics, is characterized by a high level of crime, including violent crimes, theft, and drug-related offenses. There are also diverse social problems - unemployment in certain parts of the region, poverty, social conflicts, and demonstrations - which require frequent police interventions (15, 16). Social pressure, the risk of incorrect assessment of operational decisions, and media criticism increase the psychological burden and emotional responsibility (17).

Police officers in the Silesian Voivodeship are exposed to exceptionally high work intensity. In a region with a large population and developed urban infrastructure, service often includes securing mass events, such as sports events, concerts, or demonstrations. An additional problem is the lack of balance between the number of officers and the actual needs of the units. Staff shortages, lack of appropriate logistical support, and insufficient access to training increase the pressure on employees, which favors the development of occupational burnout (18).

Previous research on occupational burnout provides valuable information; however, it rarely includes a comprehensive analysis of the specific nature of uniformed services, combining psychosocial factors (e.g., mobbing, attitudes toward work) with service conditions and socio-demographic variables. Therefore, the aim of this study is to examine the relationship between the level of occupational burnout and selected psychosocial factors, as well as socio-demographic characteristics and working conditions, in a group of police officers employed in the Silesian Voivodeship - the region with the highest urbanization rate and population density in Poland.

Drawing on Hobfoll's Conservation of Resources (COR) theory, the present study conceptualizes occupational burnout as a consequence of the progressive loss of valuable resources (19). Long working hours, and weekend shifts act as significant resource drains that consume police officers' cognitive and emotional energy, thereby contributing to competing work–family demands and Work–Family Conflict. Consequently, it is hypothesized that greater exposure to overtime and weekend work is associated with higher levels of occupational burnout among police officers (H1). In contrast, support from supervisors and colleagues can be understood as an important social resource that helps officers cope with occupational demands and protects them against further resource loss. Therefore, it is hypothesized that higher levels of perceived supervisor and co-worker support are associated with lower levels of occupational burnout (H2). Pleasure and satisfaction derived from work, including satisfaction with financial rewards, may also function as protective resources by strengthening motivation and enhancing officers' capacity to cope with demanding working conditions. Accordingly, it is hypothesized that higher levels of job and financial satisfaction are associated with lower levels of occupational burnout (H3). Finally, Work–Family Conflict may represent a competing resource demand that intensifies the effects of excessive working time and contributes to resource depletion and burnout (H4).

## 2 Materials and Methods

### 2.1 Sample and procedure

The study was conducted from May to July 2025 among police officers serving in the Silesian Voivodeship. A total of 363 police officers representing 24 organizational units of the Silesian Voivodeship Police took part in this study.

Participation in this study was voluntary and anonymous. General information about the purpose of this study and informed consent was placed on the first page of the questionnaire. Only respondents who read and signed the consent form could answer additional questions. The sample was randomly selected. Sample size was calculated using the sample size calculator (calculator.net). According to the slaska.policja.gov.pl the number of policemen in Silesian Voivodeship is about 11,000 and 380 invitations were sent. Not all selected participants agreed to participate in this study. Finally, the survey was conducted among 363 respondents. The questionnaire response rate was 96%. As the sample confidence level was 95%, the maximum sampling error did not exceed 5%. Inclusion criteria consisted of active service in police units within the Silesian Voivodeship, voluntary consent for participation, and the absence of long-term work leave (e.g., health-related or unpaid leave). Authorization for the study was issued by the Regional Police Commander in Katowice. Ethical review and approval was not required for this study because in accordance with Polish law (Act of 5 December 1996 on the Professions of Physicians and Dentists (20), if a study is based on anonymous questionnaire, the consent of bioethics committee is not required. The study complied with all research standards, including the provisions of the Declaration of Helsinki.

### 2.2 Methods

The research was conducted using both paper surveys distributed by mail and an electronic form provided through internal communication channels. Two research instruments were utilized: an original survey questionnaire and Polish adaptation of the standardized Link Burnout Questionnaire (LBQ). The original questionnaire comprised a personal data section including sociodemographic information (age, sex, marital status, place of residence, length of service, type of service, rank, and work schedule) as well as 6 questions regarding a subjective assessment of workload, the presence of psychological and somatic symptoms, and perceived social support. Furthermore, the LBQ served to measure four dimensions of occupational burnout: psychophysical exhaustion, lack of engagement in relationships, lack of professional efficacy, and disillusionment with work. Polish version of the questionnaire possesses confirmed psychometric validity and reliability, with an internal consistency coefficient (Cronbach's α) at a level satisfactory for occupational burnout research (21). Responses are provided on a 6-point frequency scale (ranging from “never” to “daily”). Sten scores are interpreted as follows: 1–3 indicates low (absence of symptoms), 4–7 indicates average (possibility of difficulties), and 8–10 indicates high level of burnout.

In the current study sample (*N* = 363), internal consistency metrics were evaluated for all four subscales using both Cronbach's alpha (α) and McDonald's omega (ω). Both coefficients demonstrated adequate to strong internal consistency across all dimensions: psychophysical Exhaustion (ω = 0.75, α = 0.59), Relationship Deterioration (ω = 0.79, α = 0.71), Lack of Professional Efficacy (ω = 0.71, α = 0.42), and Disillusionment (ω = 0.82, α = 0.70). The divergence between α and ω in subscales containing reverse-coded items (particularly Lack of Professional Efficacy) is consistent with psychometric literature (22) highlighting Cronbach's alpha sensitivity to multidimensionality and item-wording effects, confirming McDonald's omega as a more robust indicator of construct reliability.

### 2.3 Statistical analysis

Data obtained during the study were subjected to statistical analysis using methods adapted to the nature of the variables. Prior to inferential testing, the normality of distribution for all four LBQ dimensions was evaluated using Shapiro-Wilk tests, visual inspection of Q-Q plots, and skewness/kurtosis indices. Visual inspection of Q-Q plots confirmed discrete, non-Gaussian distributional patterns. Consequently, non-parametric procedures were strictly applied for group comparisons and bivariate relationship analyses. Quantitative variables, such as age and length of service, were analyzed in relation to the results of individual LBQ scales using Spearman's rank correlation, allowing for an assessment of the strength and direction of these relationships. Dichotomous qualitative variables, such as sex or the presence of psychological and somatic symptoms, were compared against LBQ scores using the Mann-Whitney U test. Conversely, multi-category qualitative variables (e.g., marital status, place of residence, type of service, work schedule, and rank) were analyzed using the Kruskal–Wallis test. *Post-hoc* comparisons were conducted utilizing the Bonferroni correction. Chi-square test was applied in the analyses of relationships between qualitative variables; however, the Mann–Whitney U–test remained the predominant method due to the specific nature of the data. The impact of situational stressors on burnout dimensions was assessed using the Welch's *t*-test, supplemented by Cohen's d effect size measure. To identify independent predictors of the overall burnout level, a multiple linear regression model was constructed. The validity of the model was confirmed through an analysis of the variance inflation factor (VIF) and an assessment of the homoscedasticity of residuals, with 95% confidence intervals determined for the parameters. The level of statistical significance was set at *p* < 0.05. All statistical analyses were performed using RStudio (Version 2024.12.1 Build 563, Posit Sotfware).

## 3 Results

The average age of the police officers was 36 years (SD = 7.99), and the length of service was 11 years (*M* = 10; Q1 = 6; Q3 = 15). Most of the participants lived in cities with over 100,000 inhabitants and were married. Approximately 60% of the police officers had children, most often one or two (Table 1).

**Table 1:** Characteristics of the study group (*N* = 363)

Variable	Total *N* = 363	Men *N* = 300	Women *N* = 63
Age (years)	36 ± 7.99	36 ± 7.82	37 ± 8.71
Place of residence			
Rural area	113 (31.13%)	95 (31.67%)	18 (28.57%)
City up to 100.000 inhabitants	91 (25.07%)	76 (25.33%)	15 (23.81%)
City over 100.000 inhabitants	159 (43.80%)	129 (43.00%)	30 (47.62%)
Marital status			
Single	112 (30.85%)	87 (29.00%)	25 (39.68%)
Married	225 (61.98%)	197 (65.67%)	28 (44.44%)
Widowed	3 (0.83%)	2 (0.67%)	1 (1.59%)
Divorced	23 (6.34%)	14 (4.67%)	9 (14.29%)
Children			
0	143 (39.39%)	120 (40.00%)	23 (36.51%)
1	98 (27.00%)	79 (26.33%)	19 (30.16%)
2	105 (28.93%)	87 (29.00%)	18 (28.57%)
3 or more	17 (4.68%)	14 (4.67%)	3 (4.76%)
Length of service as a police officer (years)	11 ± 6.81	11 ± 6.67	12 ± 7.38

Mean value ± standard deviation; *n* (%).

The most numerous group in the study consisted of police officers in preventive service and officers with the ranks of aspirant and sergeant. Most of respondents worked 40–50 h per week and had 2 days off from work. Over half of police officers reported working on weekends, and nearly 3/4 performed night duties, mostly occasionally (Table 2).

**Table 2:** Employment characteristics in the study group (*N* = 363).

Variable	Total *n* = 363	Men *n* = 300	Women *n* = 63
Weekly working hours			
Less than 40 h	1 (0.28%)	1 (0.33%)	0 (0.00%)
40 h	178 (49.04%)	141 (47.00%)	37 (58.73%)
41–50 h	163 (44.90%)	141 (47.00%)	22 (34.92%)
Over 50 h	21 (5.79%)	17 (5.67%)	4 (6.35%)
Days off per week			
1 day	53 (14.60%)	46 (15.33%)	7 (11.11%)
2 days	293 (80.72%)	240 (80.00%)	53 (84.13%)
3 days	15 (4.13%)	12 (4.00%)	3 (4.76%)
4 or more days	2 (0.55%)	2 (0.67%)	0 (0.00%)
Weekend work			
Regularly	163 (44.90%)	141 (47.00%)	22 (34.92%)
Sometimes	193 (53.17%)	153 (51.00%)	40 (63.49%)
Never	7 (1.93%)	6 (2.00%)	1 (1.59%)
Night work			
Regularly	65 (17.91%)	58 (19.33%)	7 (11.11%)
Sometimes	203 (55.92%)	168 (56.00%)	35 (55.56%)
Never	54 (14.88%)	41 (13.67%)	13 (20.63%)
Sometimes as overtime work	41 (11.29%)	33 (11.00%)	8 (12.70%)
Type of service			
Preventive	241 (66.39%)	204 (68.00%)	37 (58.73%)
Criminal	106 (29.20%)	83 (27.67%)	23 (36.51%)
Supportive	16 (4.41%)	13 (4.33%)	3 (4.76%)
Rank			
Police constable/senior constable	37 (1.19%)	26 (8.67%)	11 (17.46%)
Sergeant/senior sergeant/staff sergeant	138 (38.02%)	125 (41.67%)	13 (20.63%)
Junior aspirant/aspirant/senior aspirant/staff aspirant	155 (42.70%)	128 (42.67%)	27 (42.86%)
Sub-commissioner/commissioner/chief commissioner	26 (7.16%)	16 (5.33%)	10 (15.87%)
Sub-inspector/junior inspector/inspector	7 (1.93%)	5 (1.67%)	2 (3.17%)

*n* (%).

**Figure 1:**
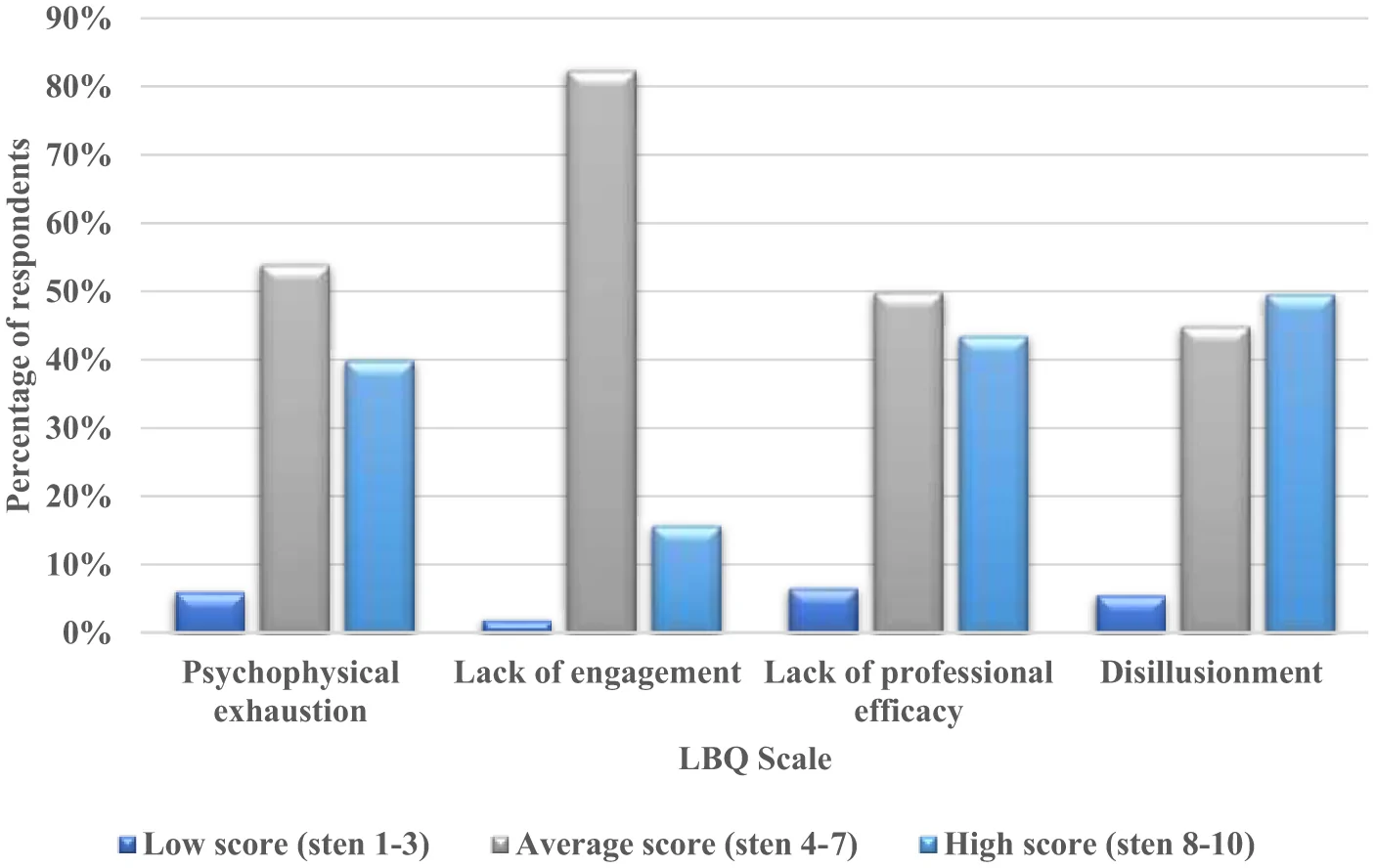
Burnout among police officers in Silesian Voivodeship measured with LBQ scale (*N* = 363).

Most officers obtained average scores in the dimension of lack of engagement in relationships (299 individuals, 82.4%), while high levels of psychophysical exhaustion and disillusionment with work were found in 145 (39.9%) and 180 (49.6%) of the participants, respectively. Nearly half of the respondents obtained high scores on the lack of professional efficacy scale (158 individuals, 43.5%). Low scores occurred relatively rarely across all scales (Figure 1).

*Post-hoc* analysis revealed significant differences in the levels of psychophysical exhaustion, lack of engagement in relationships, and disillusionment with work measured by the LBQ scale between categories of variables related to working time and the organization of leisure time. Individuals working 41–50 h per week obtained higher scores in Scale 1 (psychophysical exhaustion) and Scale 2 (lack of engagement in relationships) compared to those working 40 h per week (*p* < 0.001). Similarly, employees regularly performing duties on weekends were characterized by higher values in Scales 1, 2, and 4 (disillusionment with work) compared to those working rarely on weekends or not at all (*p* < 0.01). The number of days off also differentiated the burnout level. Individuals with only 1 day off obtained higher LBQ scores than those with 2 days off (*p* < 0.01). Furthermore, it was shown that the place of residence influenced differences in Scale 2; individuals living in cities with over 100,000 inhabitants achieved higher scores in this scale than those living in rural areas (*p* < 0.05). Detailed data are presented in Table 3.

**Table 3:** Results of Dunn's *Post-Hoc* test for significant intergroup comparisons in LBQ results (after bonferroni correction).

LBQ scale	Variable	Comparison group 1	Comparison group 2	Group 1 Me (IQR)	Group 2 Me (IQR)	*Z*	*p*
Exhaustion	Weekly working hours	40 h	41–50 h	20 (16–24)	23 (19–26.5)	−3.93	< 0.001
	Days off per week	1 day	2 days	24 (20–30)	21 (16–25)	3.92	< 0.001
	Weekend work	Yes, sometimes	Yes, regularly	20 (16–24)	22 (19–27)	−3.72	< 0.001
	Weekend work	No	Yes, regularly	16 (15.5–18)	22 (19–27)	−2.76	0.017
Lack of engagement	Working hours	40 hours	41-50 hours	21 (18–24)	23 (20–26)	−3.87	< 0.001
	Weekend work	No	Yes, regularly	17 (14.5–18)	23 (20–26)	−3.30	0.003
	Days off per week	1 day	2 days	25 (21–26)	22 (19–25)	3.22	0.008
	Weekend work	Yes, sometimes	Yes, regularly	22 (19–25)	23 (20–26)	−2.67	0.023
	Place of residence	City > 100,000 inhabitants	Rural area	23 (20–26)	21 (19–24)	2.66	0.024
	Weekend work	No	Yes, regularly	17 (14.5–18)	23 (20–26)	−2.58	0.03
Disillusionment	Weekend work	Yes, sometimes	Yes, regularly	20 (13–23)	21 (17–26)	−3.39	0.002
	Days off per week	1 day	2 days	22 (19–24)	20 (14–24)	2.74	0.037

The analysis showed that the strongest and most consistent differences between groups concerned the lack of team support. This factor was associated with a markedly higher level of psychophysical exhaustion (*d* = 0.71) and disillusionment with work (*d* = 0.65), as well as moderate differences in lack of engagement in relationships and lack of professional efficacy. Work-Family Conflict (WFC) was also significantly associated with higher levels of psychophysical exhaustion and disillusionment with work, with moderate effect sizes (*d* = 0.56–0.60). In contrast to the above factors, lack of family support did not significantly differentiate the level of any of the analyzed burnout dimensions (Table 4).

**Table 4:** Impact of situational stressors on the level of occupational burnout.

Stressor	Exhaustion		Lack of engagement		Lack of efficacy		Disillusionment	
	*t*	*d*	*t*	*d*	*t*	*d*	*T*	*d*
Lack of team support	5.74	0.71^***^	3.27	0.41^**^	2.09	0.26^*^	5.36	0.65^***^
Work-family conflict	5.07	0.56^***^	2.51	0.27^*^	1.92	0.21 (*ns*)	5.51	0.60^***^
Lack of family support	1.94	0.36 (*ns*)	1.61	0.38 (*ns*)	0.03	0.01 (*ns*)	1.13	0.23 (*ns*)

^*^p < 0.05; ^**^p < 0.01; ^***^p < 0.001.

Analysis using the Mann–Whitney *U*-test revealed statistically significant differences in the level of occupational burnout between individuals reporting the occurrence of specific symptoms and those in whom they did not occur. The strongest differentiation (*p* < 0.001) was observed in the dimension of lack of engagement. For this scale, the presence of each analyzed symptom was associated with significantly higher burnout scores. A similar trend, with the exception of sleep problems, was noted in the area of disillusionment with work. Notably, the dimension of lack of professional efficacy was the least differentiated by the examined somatic and psychological symptoms (Table 5). Significant differences in this scale occurred only in the cases of lack of motivation (*p* < 0.05) and lowered self-esteem (*p* < 0.001), while for other variables (e.g., irritability, sleep problems), the results proved statistically nonsignificant (ns). Detailed results are presented in Table 5.

**Table 5:** Relationship between the occurrence of symptoms and the level of occupational burnout measured by the LBQ scale.

Variable	Exhaustion			Lack of engagement			Lack of efficacy			Disillusionment		
	No Me (IQR)	Yes Me (IQR)	U (*p*)	No Me (IQR)	Yes Me (IQR)	U (*p*)	No Me (IQR)	Yes Me (IQR)	U (*p*)	No Me (IQR)	Yes Me (IQR)	U (*p*)
Lack of motivation	18 (15–22)	24 (21–28)	7566^**^	21 (18–23)	24 (21–26)	955.5^***^	14 (10–19)	15 (12–18)	14294^*^	18 (12–21)	23 (19–28)	8482^**^
Lack of interest in duties	20 (16–24)	25 (22–29)	6008^**^	21 (18–25)	25 (21–27)	7775^***^	14 (11–19)	15 (12–19)	10565 ns	19 (14–22.8)	24 (20–30)	5959^**^
Cynical approach toward others	20 (16–24)	25 (22–29)	5008^**^	22 (19–25)	25 (21–27.5)	5855^***^	14 (11–19)	14 (12–17)	9297 ns	20 (14–23)	24 (19.5–29)	5766.5^**^
More frequent illnesses, infections	21 (16–25)	24 (21–28)	3673^**^	22 (19–25)	24 (21–26)	4779.5^*^	14 (11–18)	16 (12–19)	5165.5 ns	20 (14.2–24)	23 (19–26)	4021^**^
Irritability	20 (15–23)	23 (19–27)	10372^**^	21 (18–24)	24 (21–26)	11107.5^***^	14 (11–19)	14 (11–18)	15819 ns	19 (14–23)	22 (17–27)	11494^**^
Somatic symptoms	21 (16–24)	25 (21–30)	5574^**^	22 (19–25)	24 (21–26)	7545^**^	14 (11–18)	15 (12–19)	8903 ns	20 (14.2–24)	22 (17–25)	7664^**^
Lowered self-esteem	21 (16–24)	25.5 (22–29)	4395^**^	22 (19–25)	24 (21–26)	5683.5^**^	14 (11–18)	17 (14.2–19.8)	5322^**^	20 (14–24)	23 (20–29)	4985^**^
Sense of lack of professional efficacy	20 (16–24)	24 (21–28)	6851^**^	22 (18.5–25)	24 (21–27)	7818.5^***^	14 (11–18)	15.5 (12–19)	9326 ns	19 (13–23)	23.5 (20–30)	5835.5^**^
Sleep problems	20 (15–23)	24 (19–27)	10035^**^	21 (18–25)	23 (21–26)	12305.5^**^	14 (11–19)	14 (12–18)	14886 ns	20 (14–24)	21 (17–24)	13823 ns
Difficulty concentrating	20 (16–24)	23.5 (21–27.2)	815^**^	22 (18–25)	23 (21–26)	9893.5^***^	14 (11–19)	15 (12–18)	12291 ns	19 (14–23)	23 (19–27)	8694.5^**^
Difficulty making decisions	21 (16–25)	26 (22.5–31)	2496^**^	22 (19–25)	24 (22–26.5)	3441.5^**^	14 (11–18)	18 (15–20.5)	3369.5^**^	20 (14.8–24)	27 (20.5–31.5)	2464.5^**^
Mood swings	21 (16–24)	23 (19–27)	9593^**^	22 (19–25)	23.5 (20.8–26)	10689.5^*^	14 (11–19)	15 (12–19)	11547 ns	20 (14–24)	21 (18–28)	9749^**^
Social withdrawal	21 (16–24)	25.5 (22–29.2)	4618^**^	22 (19–25)	25 (21.8–27)	5723^***^	14 (11–19)	14.5 (12–18)	8332 ns	20 (14–23)	24 (19–29)	5132.5^**^
Fatigue/exhaustion	19 (15–22)	24 (20–27)	8408^**^	20 (18–23.5)	24 (21–26)	1015.5^***^	14 (11–19)	15 (12–18)	1545.5 ns	18 (12–22)	22 (18–26.2)	10113^**^

Data are presented as Median (interquartile range, Q1–Q3) for raw LBQ subscale scores. “No” indicates absence of the symptom, “Yes” indicates presence of the symptom. *U* = Mann–Whitney U test statistic; *p* = statistical significance level (^*^p < 0.05, *p* < 0.01, *p* < 0.001, ns, not significant).

Correlation analysis revealed that the strongest and most consistent relationships with all dimensions of burnout concerned pleasure derived from work (Table 6). This resource was strongly negatively associated with both psychophysical exhaustion (*r* = −0.63) and disillusionment with work (*r* = −0.69), and moderately associated with lack of engagement in relationships and lack of professional efficacy (*p* < 0.001). A similar, though slightly weaker pattern of relationships was observed for the sense of being appreciated and willingness to choose the profession again, which showed significant negative correlations with the key components of burnout, particularly with psychophysical exhaustion and disillusionment with work ( −0.63, ≤ *r* ≤ −0.41)). Among negative factors, poor workplace relationships correlated positively with all dimensions of burnout, most strongly with disillusionment with work (*r* = 0.41) and lack of professional efficacy (*r* = 0.39). Pay satisfaction showed weaker but significant protective relationships toward psychophysical exhaustion and disillusionment with work, but was not associated with lack of professional efficacy.

**Table 6:** Relationship between personal resources and occupational burnout subscales.

Resource/attitude	Exhaustion (*r*)	Lack of engagement (*r*)	Lack of efficacy (*r*)	Disillusionment (*r*)
Pleasure derived from work	−0.63^***^	−0.33^***^	−0.30^***^	−0.69^***^
Sense of being appreciated	−0.51^***^	−0.31^***^	−0.27^***^	−0.50^***^
Career choice again	−0.41^***^	−0.32^***^	−0.29^***^	−0.63^***^
Stress coping	−0.40^***^	−0.11^*^	−0.41^***^	−0.33^***^
Poor workplace relationships	0.37^***^	0.14^**^	0.39^***^	−041^***^
Pay satisfaction	−0.29^***^	−0.23^***^	0.05 (*ns*)	−0.32^***^

^*^p < 0.05; ^**^p < 0.01; ^***^p < 0.001.

The overall multiple linear regression model was statistically significant, F(16, 346) = 15.54, *p* < 0.001, accounting for 41.8% of the total variance in psychophysical exhaustion (*R*^2^ = 0.418, Adjusted *R*^2^ = 0.391). The model indicated that pleasure derived from work remained the strongest protective factor against exhaustion (*B* = −4.88; β = −0.30; *p* < 0.001). An increase in this resource was associated with a clear reduction in the level of exhaustion, regardless of the presence of numerous organizational stressors and demographic variables. A significant protective influence was also shown by the willingness to choose the profession again (*B* = −2.89; β = −0.21; *p* < 0.001), which emphasizes the importance of a positive attitude toward one's own career path.

Among the risk factors, poor workplace relationships (*B* = 2.97; β = 0.15; *p* = 0.001) and experience of workplace bullying/mobbing (*B* = 1.34; β = 0.10; *p* = 0.028) were of particular importance, as they were associated with a significantly higher level of exhaustion after accounting for other variables in the model. Additionally, significant but weaker protective effects were noted for the sense of being appreciated (*B* = −1.74; β = −0.11; *p* = 0.026) and stress coping (*B* = −1.68; β = −0.09; *p* = 0.040). Detailed data are presented in Table 7.

**Table 7:** Predictors of exhaustion level in a multiple linear regression model.

Independent variable	*B*	SE	β	*t*	*P*	VIF
Pleasure derived from work	−4.88	0.90	−0.30	−5.40	< 0 .001	2.24
Career choice again	−2.89	0.69	−0.21	−4.20	< 0.001	1.77
Poor workplace relationships	2.97	0.89	0.15	3.35	0.001	1.47
Experience of workplace bullying/mobbing	1.34	0.61	0.10	2.20	.028	1.64
Sense of being appreciated	−1.74	0.78	−0.11	−2.23	.026	1.80
Stress coping	−1.68	0.82	−0.09	−2.06	.040	1.33
Work-family conflict	2.70	1.53	0.07	1.77	.078	1.20
Weekend work	1.65	1.54	0.05	1.07	.285	1.34
Night shifts	−1.64	1.94	−0.04	−0.85	.397	1.26
Length of service	−0.10	0.16	−0.04	−0.62	.533	2.74
Pay satisfaction	−0.45	0.62	−0.03	−0.73	.467	1.28
Lack of team support	0.77	1.82	0.02	0.42	.675	1.41
Sex (Female)	0.90	1.78	0.02	0.50	.614	1.03
Number of working hours	0.28	1.13	0.01	0.25	.804	1.08
Age	0.02	0.14	0.01	0.12	.908	2.83
Lack of family support	−0.00	2.88	−0.00	−0.00	.999	1.13

*R*^2^ = 0.418; “Adjusted ” *R*^2^ = 0.391; *F* (16,346) = 15.54, *p* < 0.001.

## 4 Discussion

This study provides detailed data on the mechanisms underlying the development of occupational burnout among police officers in the Silesian Voivodeship. The findings indicate that this phenomenon is both widespread and multidimensional. High levels of psychophysical exhaustion were recorded in nearly 40% of the participants, while disillusionment with work affected almost half of the respondents (49.6%). These indicators correspond with international research, suggesting that the specific nature of police service generates universal psychological costs regardless of the country (4, 23–25). However, the analysis of the present research material allows for the identification of determinants specifically relevant to the Polish context.

Many police officers serve for years under conditions of constant emotional tension and contact with human suffering (5). Long-term exposure to dramatic situations, such as domestic violence, crimes involving the use of force, traffic accidents, or sudden social crises, leads to the gradual accumulation of stress and psychological burden (26). Over time, these experiences may result in occupational burnout, characterized by a loss of empathy, emotional indifference, and a sense of helplessness toward recurring difficult events. As Oliveira (27) indicates, in high-risk professions such as police service, chronic stress is particularly intense because it is coupled with the necessity of making decisions under pressure, risk, and responsibility for lives of others. Anders et al. emphasize that multi-year exposure to such burdens leads to deep changes in the emotional and cognitive functioning of officers, increasing the risk of depersonalization and occupational burnout (28).

A detailed analysis of working conditions revealed that the key risk factor is not the mere fact of performing the service, but rather its intensity and the lack of opportunity for recovery. A statistically significant difference in the levels of exhaustion and lack of engagement was demonstrated between individuals working a standard 40-h week and those working 41–50 h per week (*p* < 0.001). Notably, the linear model showed that weekend work, while strongly differentiating burnout levels in univariate analyses, loses significance in the multivariate model in favor of “soft” factors such as satisfaction or relationships. This suggests that the temporal workload serves as a background for burnout-creating fertile ground upon which the syndrome develops when psychological resources are lacking.

The impact of restricted days off from work also warrants attention. Officers with only 1 day off per week exhibited significantly higher exhaustion indicators (*Z* = 3.92; *p* < 0.001). This confirms Hobfoll's Conservation of Resources (COR) theory and hypothesis H1. According to COR theory, a chronic deficit of rest limits an individual's ability to rebuild resources, which in the context of high-risk professions leads to a “loss spiral” and a permanent reduction in adaptive capacity (29, 30). In practice, this means that not only the intensity of service but also the lack of adequate recovery time is a key factor deteriorating the psychophysical wellbeing of officers. These results are consistent with observations from research on police officers which showed that insufficient rest during time off work facilitates the development of emotional burnout and a decline in psychological resilience (31).

Similar relationships are confirmed by authors worldwide. Peterson et al. (32) demonstrated that irregular working hours and variable shift rhythms among police officers significantly increase burnout levels through sleep disturbances and chronic rest deficits. In studies by Sreedisha and Celina (33), it was confirmed that the inability to effectively regenerate outside of service correlates with reduced psychological resilience and an increased tendency to develop burnout. Furthermore, Mehta (34) emphasizes that under conditions of high stress and low sleep quality, officers enter the phase of emotional exhaustion more quickly. Notably, Burlakova and Melnychuk (35) suggest that an excessive number of working hours without parallel psychological support leads to a permanent decline in wellbeing and increases the level of burnout. In work of Jin Gao et al. workload of prison police workers had the strongest correlation with burnout, followed by the physical condition and sense of rejection (36).

One of the most intriguing results of the study concerns the source of social support. According to this study a lack of team support is a powerful stressor influencing exhaustion and disillusionment. In comparison, the lack of family support proved to be a statistically insignificant factor for any of the analyzed burnout dimensions, fully supporting hypothesis H2.

Similar conclusions were drawn by Lambert et al. (37), indicating that support from supervisors and colleagues constitutes one of the most important protective factors against burnout, and its absence intensifies the effects of Work-Family Conflict (WFC). This support is not limited to instrumental help but includes a sense of community and understanding which are crucial for maintaining emotional stability in uniformed environments. Wolter et al. (38) add that a high level of support at work increases professional engagement and the sense of self-efficacy, providing a counterweight to chronic operational stress.

Conversely, the results regarding the marginal role of family support confirm the observations of Singh and Nayak (39), who noted that while family support can reduce emotional tension, its impact on the level of burnout itself is much weaker than organizational support. In the police environment, family relationships are not perceived as a source of substantive understanding of the difficulties of service. This is also confirmed by more recent research by Kaur (40), indicating that positive family relationships support the general emotional resilience of officers, yet the organizational climate and team culture have a decisive significance in burnout prevention.

It should be emphasized that the strong influence of conflict between work and home fits into the model of indirect action of occupational stressors described by Griffin and Sun (41), according to which an imbalance between private and professional life can lead to an escalation of emotional tension, especially when support from colleagues is lacking (confirming hypothesis H4). Consequently, it is the team, not the family, that becomes the primary buffer for officers protecting them against the psychological effects of overload and the loss of meaning in work.

The analysis of accompanying symptoms proves that burnout among Silesian police officers is not a purely emotional phenomenon but has a strong somatic component. Exhaustion correlated at a very high level of significance (*p* < 0.001) with sleep problems, irritability, and somatic symptoms. Similar results were obtained in international studies, where somatic symptoms such as sleep disturbances or chronic fatigue co-occurred with occupational burnout in officers (4, 42).

An interesting exception is the dimension of lack of professional efficacy, which proved to be the least sensitive to somatic symptoms. This means that an officer can be physically exhausted and suffer from insomnia yet still rate their professional competencies highly. This is a potentially dangerous adaptive mechanism, maintaining high professional self-esteem at the expense of physical health, which in the long run can lead to sudden health breakdowns (25, 43).

The literature on the subject indicates that similar mechanisms have been observed in studies on occupational stressors and burnout in groups of paramedics and firefighters-groups in which the pressure to maintain effectiveness despite exhaustion is part of the professional ethos (28, 44). These results confirm the necessity of introducing preventive interventions among members of uniformed services, targeted not only at the emotional sphere but also at the physical effects of chronic occupational stress.

The most important conclusion drawn from the study is the hierarchy of protective factors identified in the regression model (Table 7). Pleasure derived from work (understood as internal satisfaction/a sense of meaning) proved to be the strongest independent predictor protecting against exhaustion. Its influence was more than twice as strong as the influence of, for example, the sense of being appreciated or stress coping. Moreover, pay satisfaction, although it correlated with burnout, ceased to be a significant predictor in the regression model. Thus, hypothesis H3 was only partially supported: in the face of difficult service conditions, financial factors are of secondary importance compared to internal factors (motivation) and relational ones.

On the other hand, “poor workplace relationships” and “experience of workplace mobbing” were identified as independent, strong risk factors. The literature confirms that negative interpersonal relationships and psychological violence in the workplace significantly increase the risk of occupational burnout in uniformed services. Studies in Australia have shown that mobbing and a hostile work environment among officers lead to reduced job satisfaction, increased absenteeism, and intentions to leave the service (45). Similar relationships were noted in a study conducted in Africa, where mobbing and excessive organizational demands were strongly linked to occupational stress symptoms and reduced psychological wellbeing of police officers (46). Furthermore, another analysis indicated that police mobbing can lead to the intensification of occupational burnout, particularly in officers with low levels of psychological capital -the ability for self-regulation and emotional resilience (47). It has been previously established that mobbing in high-stress environments, such as the police, fire service, or rescue, is not only frequent but also strongly associated with psychosomatic symptoms and a reduction in the sense of professional efficacy (48).

It should be noted that the studied group was characterized by an overrepresentation of individuals from large urban centers (43.8% from cities over 100,000), which is specific to the densely populated Silesian Voivodeship. This may affect the higher level of operational stress compared to units in other regions of the country. Attention is also drawn to the risk of social desirability bias. The assessment of psychosocial factors was based on self-declarations by the participants, which in the case of police officers could involve a tendency to provide answers more in line with social or professional expectations, rather than fully reflecting the actual level of difficulties experienced. Fear of evaluation, stigmatization, or potential professional consequences might have influenced the underreporting of occupational burnout symptom intensity despite the anonymity of the study. Furthermore, the study was cross-sectional, which does not allow for an assessment of burnout dynamics over time. Future research projects should include longitudinal studies, especially in the context of the changing police workforce and the influx of young officers (Generation Z), whose expectations of service may differ from older cohorts.

The regional context of the Silesian Voivodeship shows that occupational burnout among police officers is a multidimensional phenomenon resulting from the interaction of organizational, social, and individual factors. High operational demands, limited support resources, and pressure resulting from social and media expectations create an environment conducive to chronic stress and emotional burden. In an era of dynamic changes in the structure of the Police, new technological challenges, an increasing number of interventions, and rising social expectations, the problem of occupational burnout among officers gains particular relevance. It constitutes not only a research problem but also a practical challenge for the public safety system, requiring the implementation of effective preventive, organizational, and psychological actions.

## Data Availability

The raw data supporting the conclusions of this article will be made available by the authors, without undue reservation.
